# The Role of Zinc in Copper Homeostasis of *Aspergillus fumigatus*

**DOI:** 10.3390/ijms21207665

**Published:** 2020-10-16

**Authors:** Suzie Kang, Hyewon Seo, Hee-Soo Moon, Joon-Ho Kwon, Yong-Sung Park, Cheol-Won Yun

**Affiliations:** School of Life Sciences and Biotechnology, Korea University, Anam-dong, Sungbuk-gu, Seoul 02841, Korea; sthe327@korea.ac.kr (S.K.); hyewon330@korea.ac.kr (H.S.); heesoomoon@korea.ac.kr (H.-S.M.); joonhokwon@korea.ac.kr (J.-H.K.); dcomtrue@korea.ac.kr (Y.-S.P.)

**Keywords:** copper, zinc, CtrC, ZafA, *Aspergillus fumigatus*

## Abstract

Copper is an essential metal ion that performs many physiological functions in living organisms. Deletion of *Afmac1,* which is a copper-responsive transcriptional activator in *A. fumigatus*, results in a growth defect on aspergillus minimal medium (AMM). Interestingly, we found that zinc starvation suppressed the growth defect of the *Δafmac1* strain on AMM. In addition, the growth defect of the *Δafmac1* strain was recovered by copper supplementation or introduction of the *CtrC* gene into the *Δafmac1* strain. However, chelation of copper by addition of BCS to AMM failed to recover the growth defect of the *Δafmac1* strain. Through Northern blot analysis, we found that zinc starvation upregulated *CtrC* and *CtrA2*, which encode membrane copper transporters. Interestingly, we found that the conserved ZafA binding motif 5′-CAA(G)GGT-3′ was present in the upstream region of *CtrC* and *CtrA2* and that mutation of the binding motif led to failure of ZafA binding to the upstream region of *CtrC* and upregulation of *CtrC* expression under zinc starvation. Furthermore, the binding activity of ZafA to the upstream region of *CtrC* was inversely proportional to the zinc concentration, and copper inhibited the binding of ZafA to the upstream region of *CtrC* under a low zinc concentration. Taken together, these results suggest that ZafA upregulates copper metabolism by binding to the ZafA binding motif in the *CtrC* promoter region under low zinc concentration, thus regulating copper homeostasis. Furthermore, we found that copper and zinc interact in cells to maintain metal homeostasis.

## 1. Introduction

Transition metals, such as copper and zinc, are essential elements for living organisms and have important roles in the virulence of pathogenic microbes [1,2,3]. Metals are major components of life and are both vital and toxic for living organisms [4]. Accumulation of divalent metals leads to production of hydroxyl radicals, which are a type of ROS, through the Fenton reaction [5,6], and ROS can damage proteins, lipids, and DNA. Therefore, metal homeostasis is strictly regulated in living cells. Metal homeostasis has been studied in yeast and many other microorganisms as model systems, including *Aspergillus fumigatus*. As with other pathogenic microorganisms, metal homeostasis is closely related to the virulence of *A. fumigatus* [7,8,9]. In particular, copper has an important role in the growth of conidia [10,11,12], which directly affects the virulence of *A. fumigatus*, and the copper-responsive transcription factor AfMac1 is known to be directly related to the virulence of *A. fumigatus* [11,13].

When copper is depleted, AfMac1 directly interacts with copper through its Cys-rich domain at the N-terminus of the protein [14]. AfMac1 upregulates expression of the *Ctr* family genes [14,15,16,17], which encode copper transporters to overcome copper starvation. AfMac1 has been reported to directly bind the consensus binding motif 5′-TGTGCTCA-3′ [11], which is in the promoter region of *CtrA2* and *CtrC*, to upregulate expression of the genes [11,15]. On the other hand, when the cell is overloaded with copper, the low-affinity copper-responsive transcription factor AceA is activated to promote the expression of CrdA, a metallothionein [18], to protect the cell from copper toxicity [19]. The copper concentration in the cell is also known to be controlled by expression of CrpA, a copper exporter [19,20]. CrpA is a P-type ATPase copper transporter located on the surface of the cell that pumps copper out of the cell to prevent excessive copper accumulation inside the cell. When *CrpA* is deleted, *A. fumigatus* shows a copper-sensitive phenotype [19]. 

Zinc also plays an important role in stabilizing the structure of proteins. Approximately 40% of zinc-binding proteins are known to be transcription factors with a zinc finger domain [21,22], and they play an important role as co-factors to assist enzyme functions in cells [21,23,24]. Zinc homeostasis in cells is largely controlled by two groups: the Zrt- and Irt-like protein (ZIP) transporter family and the cation diffusion facilitator (CDF) family [9,25,26]. The CDF transporter family includes MtpA and B and ZrcA, B, and C [27,28,29]. These transporters are expressed when zinc accumulates in the cell and take up the zinc into vacuoles for zinc storage to control its toxicity [19]. Conversely, when zinc is depleted, ZafA, a zinc response transcription factor, induces expression of the ZIP transporter family [30]. The transcriptional function of ZafA is regulated by direct binding of zinc [30,31]. When zinc is replete, zinc binds to ZafA to inhibit its activity [23]. However, when zinc is depleted, zinc is separated from ZafA, and ZafA activates expression of the Zrf family of zinc transporters [26,30]. In addition to the zinc concentration, Zrf family gene expression is controlled by the pH of the environment [26]. The PacC protein inhibits the function of ZafA according to pH [32]. When the environment is acidic or neutral, ZafA upregulates the expression of ZrfA and B [26]. Under a basic environment, PacC binds to the ZafA binding site to suppress the expression of ZrfB and A and upregulates the expression of ZrfC. In addition to pH, ZrfC is strongly upregulated under zinc-deficient conditions, and when *A. fumigatus* causes aspergillosis in the host, it plays an important role in zinc uptake and has a significant function in virulence [9].

*A. fumigatus* is an opportunistic pathogen that can cause fatal disease in elderly people or organ transplant patients with an impaired immune system. Transition metals, such as iron, copper, and zinc, have become important factors in preventing microbial infections in humans because the ability of infectious microorganisms to utilize metals is known to play an important role in their survival and growth [1,33]. Hosts have developed various mechanisms to separate essential nutrients from pathogens that cause infection from the source of infection [34,35]. Macrophages employ an antipathogen strategy using copper poisoning, which eliminates pathogens by triggering ROS formation through activation of NADPH oxidase [36]. According to a previous study, to protect against pathogenic bacteria, macrophages increase the expression of Ctr1, a copper transporter in bacteria, and move ATP7A, a P-type copper ATPase pump, to a phagolysosomal membrane [37,38]. Neutrophils also protect hosts from pathogens by depleting zinc through calprotectin. Calprotectin is an antimicrobial protein expressed by neutrophils, and approximately 40% of calprotectin is synthesized in neutrophils. Calprotectin inhibits the growth of pathogens by separating zinc from the pathogen [39].

Recently, we reported that iron and copper interact functionally in *A. fumigatus* and that copper regulates iron metabolism, and vice versa [40,41]. We observed that iron regulates the expression of genes related to copper metabolism, and another group reported that iron and zinc functionally interact with one another. For example, we reported that the copper-responsive transcription factor AfMac1 regulates the expression of genes involved in the reductive and siderophore-mediated iron uptake system [40]. This report indicates that metals interact with each other in cells to regulate homeostasis of other metals, and this regulation is mutual. In the present study, we found that zinc regulates the expression of genes related to copper metabolism at the transcriptional level. These results support our hypothesis of mutual regulation of metal homeostasis in living organisms and provide a good model for developing antifungal drugs.

## 2. Results

### 2.1. Growth Defect of the Δafmac1 Strain was Recovered by Zinc Starvation

**Figure 1:**
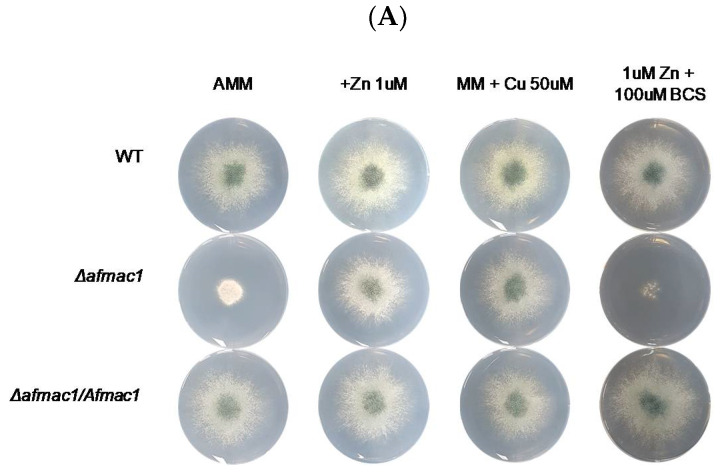

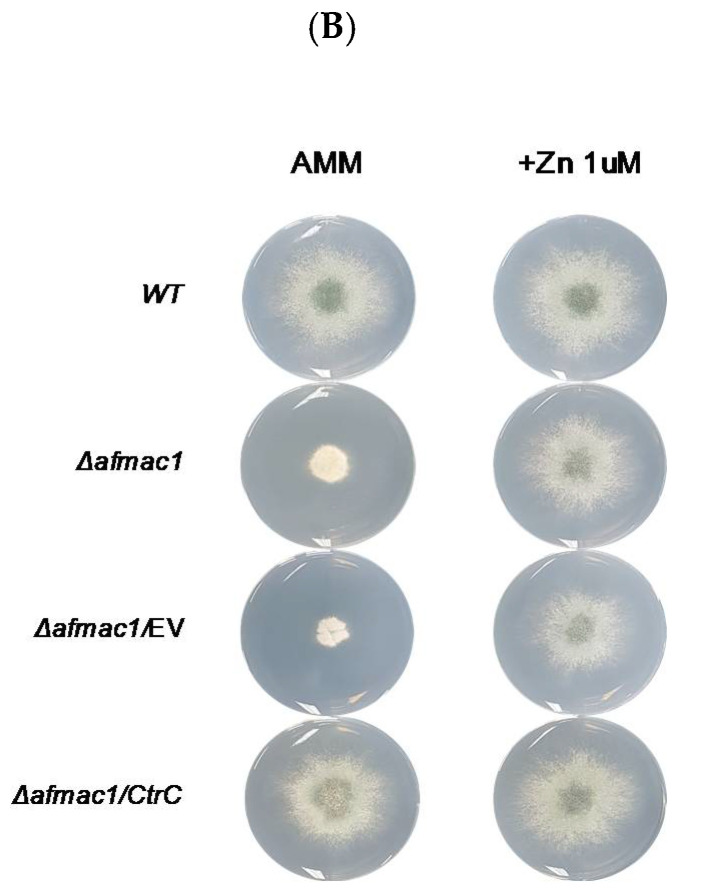
Zinc starvation suppressed the growth defect of the *AfMac1* deletion mutant. To investigate the effect of zinc on growth of the *AfMac1* deletion mutant, a spotting assay was performed with the wild-type, *Δafmac1*, and *AfMac1* complemented strains of *A. fumigatus*. (**A**) Conidia (10 × 10^5^) of the indicated strain were spotted on AMM (77 mM zinc), zinc-starved (1 µM zinc) medium, copper-supplemented (50 mM copper) medium, or BCS-supplemented medium and incubated for 48 h at 37 °C. (**B**) Additionally, *CtrC* was introduced into the *Δafmac1* strain, and the effect of *CtrC* was investigated. Conidia (10 × 10^5^) of the indicated strain were spotted on AMM or zinc-starved (1 µM zinc) medium and incubated for 48 h at 37 °C.

Zinc is an essential trace element and performs various physiological functions in living cells. Previously, we found that copper regulates iron metabolism [40], and in this report, we sought to understand the interaction between zinc and copper metabolism. To investigate the effect of zinc on copper metabolism in *A. fumigatus*, the effect of zinc on growth of the *Δafmac1* strain was investigated. AfMac1 is a copper-responsive transcription activator that regulates gene expression of the high-affinity copper transporter encoding genes *CtrC* and *CtrA2* in *A. fumigatus*. The *Δafmac1* strain showed slow growth on standard AMM in a copper-dependent manner, and copper supplementation recovered the growth defect of the *Δafmac1* strain. Interestingly, we found that the *Δafmac1* strain showed a normal growth rate when starved of zinc but not on standard AMM, as shown in Figure 1A. The complemented strain of *Δafmac1/AfMac1* showed the same growth rate as the wild-type strain on AMM. To confirm whether the growth defect resulted from copper deficiency, 50 µM copper was added exogenously to the medium. The growth defect of *Δafmac1* was recovered by high copper concentration, and this result indicates that the growth defect was a result of copper deficiency. To further investigate the reason why the growth defect was suppressed by zinc starvation, BCS was added to the medium. As shown in Figure 1A, growth recovery by zinc starvation failed when BCS was added to the medium. This result indicates that growth recovery by zinc starvation results from the ability to utilize copper in the medium, and when copper is chelated by BCS, the growth defect fails to recover. This result was confirmed by introduction of *CtrC* into the *Δafmac1* strain, as shown in Figure 1B. CtrC is a major high-affinity copper transporter in *A. fumigatus,* and high copy expression of *CtrC* suppressed the growth defect of the *Δafmac1* strain in AMM. These results indicate that the phenotype of growth recovery of the *Δafmac1* strain by zinc starvation is the result of increased uptake of copper.

### 2.2. The Expression of Genes that Encode Copper Transporters Was Regulated by Zinc

**Figure 2:**
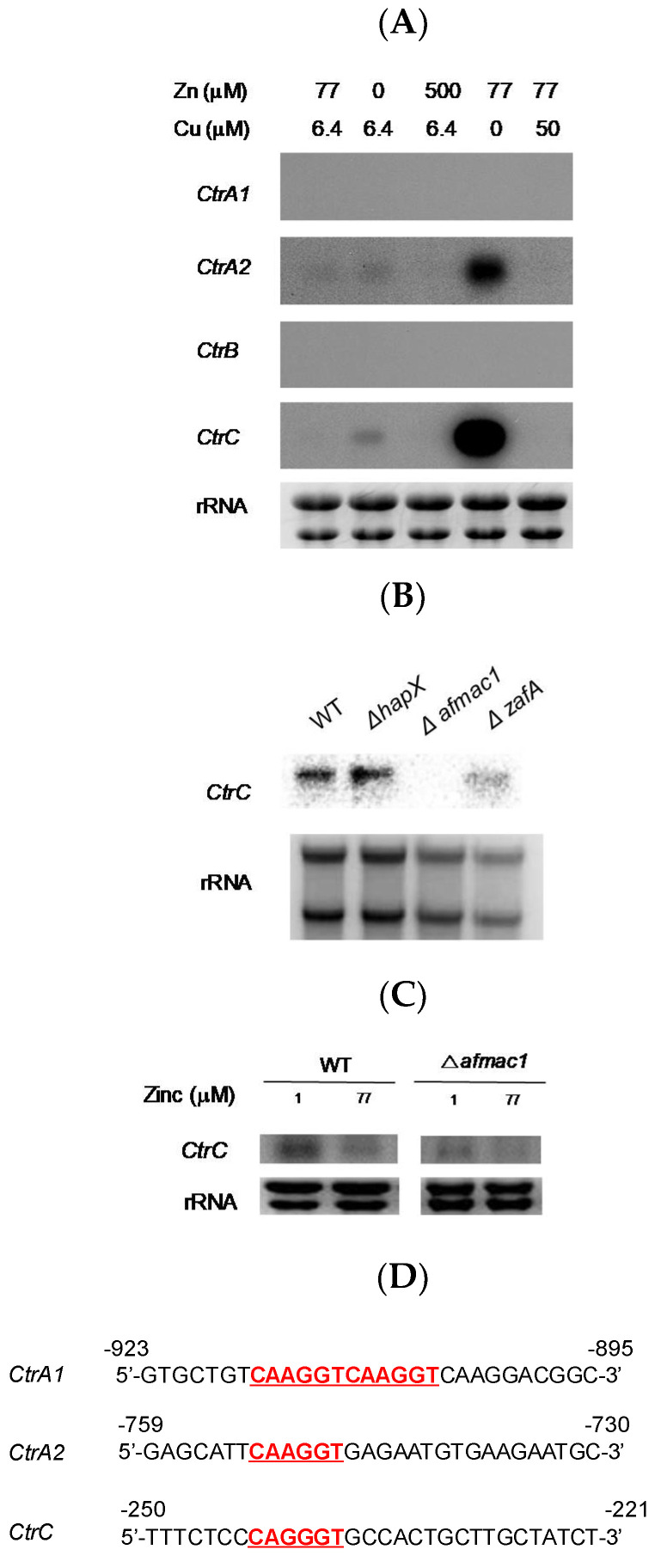
*CtrC* expression was regulated by zinc and ZafA. To investigate the effect of zinc on the expression of *Ctr* genes, Northern blotting was performed. (**A**) Cells were cultured in different media containing the indicated metal concentrations until mid-log phase. Total RNA was extracted, and then Northern blotting was performed (77 µM zinc and 6.4 µM copper indicate AMM, 0 µM zinc indicates zinc starvation medium, 500 µM zinc indicates zinc sufficient medium, and 50 µM copper indicates copper sufficient medium). The probes targeted *CtrA1, CtrA2, CtrB*, and *CtrC*, which are members of the *Ctr* gene family. (**B**) The effect of different transcription factors involved in metabolism of diverse metals on the expression CtrC was investigated. The deletion mutant of the indicated gene was cultured in AMM, and total RNA was extracted. HapX is an iron-regulated transcriptional activator. (**C**) The effect of zinc on the expression of *CtrC* was evaluated in the *Δafmac1* strain. The wild-type and *Δafmac1* strains were cultured in the indicated zinc concentration until mid-log phase, and total RNA was extracted (1 µM zinc indicates zinc starvation media, and 77 µM zinc indicates AMM). (**D**) Sequencing analysis of the promoter region of the *Ctr* gene family was performed. The conserved ZafA binding motif 5′-CAA(G)GGT-3′ was found in the promoter regions of *CtrA1*, *CtrA2*, and *CtrC* but not in that of CtrB.

To investigate the reason why zinc starvation suppresses the growth defect of the *AfMac1* deletion mutant, Northern blot analysis was performed. As shown in Figure 1B, exogenous introduction of *CtrC* recovered the growth defect of the *AfMac1* deletion mutant, and this result suggests the possibility of *CtrC* expression involvement in recovery of the *AfMac1* deletion mutant. Four putative copper transporters are reported in the *A. fumigatus* genome [14,15], and we performed Northern blot analysis to investigate the expression of the four *Ctr* genes in response to zinc utilization. As shown in Figure 2A, *CtrA2* and *CtrC* expression was upregulated under zinc starvation conditions, and *CtrC* expression was more upregulated by zinc starvation than *CtrA2*. A previous report showed that *CtrC* expression is not detected when *AfMac1* is deleted because AfMac1 is a copper-responsive transcription activator that upregulates the expression of *CtrC* under low copper concentration [15].

Furthermore, the effect of iron and zinc on the expression of *CtrC* was investigated in the *HapX* and *ZafA* deletion strains. HapX and ZafA are transcriptional activators that regulate expression of the genes involved in iron and zinc metabolisms, respectively. As shown in Figure 2B, *CtrC* expression was detected in the wild-type and *HapX* deletion mutant but not in the *AfMac1* deletion mutant. Interestingly, deletion of *ZafA* resulted in downregulation of *CtrC*. As shown in Figure 1, the *AfMac1* deletion strain showed a growth defect on AMM, and zinc starvation recovered the growth defect. We investigated the expression of *CtrC* in the *AfMac1* deletion strain after zinc starvation. As shown in Figure 2C, *CtrC* expression was very low when the cells were cultured in AMM. However, the expression of *CtrC* was increased under zinc starvation conditions. We found that ZafA and zinc regulate *CtrC* gene expression, and we performed sequence analysis of *Ctr* genes to determine whether a ZafA binding motif is present on the promoter region of *Ctr* genes to understand how zinc and ZafA regulate the expression of *Ctr* genes. As shown in Figure 2D, the conserved ZafA binding motif 5′-CAA(G)GGT-3′ was found in the promoter regions of *CtrA1*, *CtrA2*, and *CtrC* but not in that of *CtrB.* However, the ZafA binding motifs in *CtrA1* and *CtrA2* were distant from the transcription start site, while the ZafA binding motif in CtrC was located in the −243 region from the *CtrC* upstream region and thus was closer than in the other *Ctr* genes.

### 2.3. ZafA Regulates the Expression of Ctr Genes via the ZafA Binding Motif

**Figure 3:**
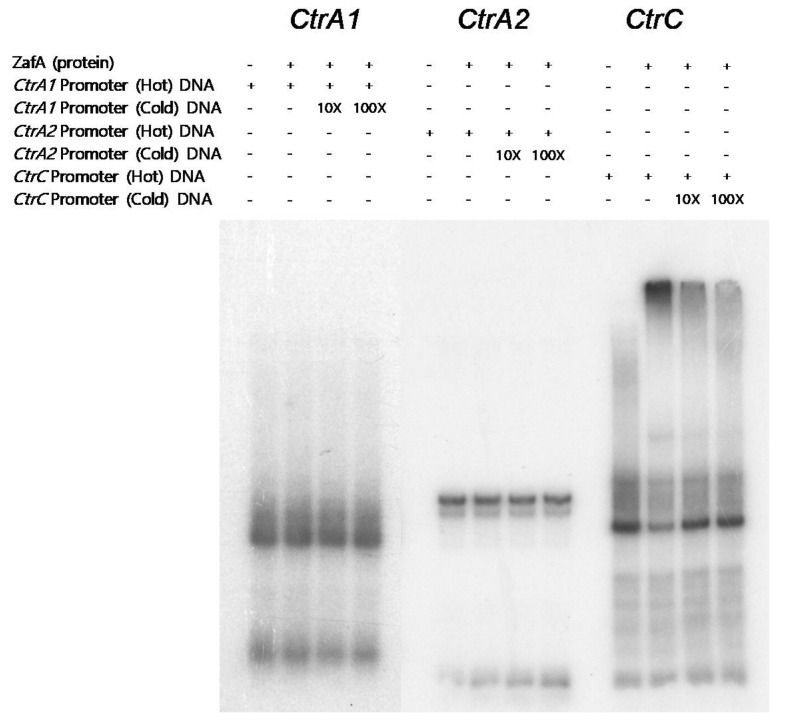
ZafA directly binds to the promoter region of CtrC. The conserved ZafA binding motif 5′-CAA(G)GGT-3′ was found in the promoter regions of *CtrA1, CtrA2*, and *CtrC*. To investigate the role of ZafA in the expression of *Ctr* genes, an EMSA experiment was performed with DNA fragments that contained the conserved ZafA binding motif. Briefly, 118, 114, and 128 bp DNA fragments of the promoter regions of *CtrA1, CtrA2*, and *CtrC*, respectively, which contain the conserved ZafA binding motif, were amplified. P^32^-labeled probes (hot) were reacted with recombinant ZafA protein, and the reaction mixtures were separated via PAGE. Unlabeled probes (cold) were used as a competitor.

**Figure 4:**
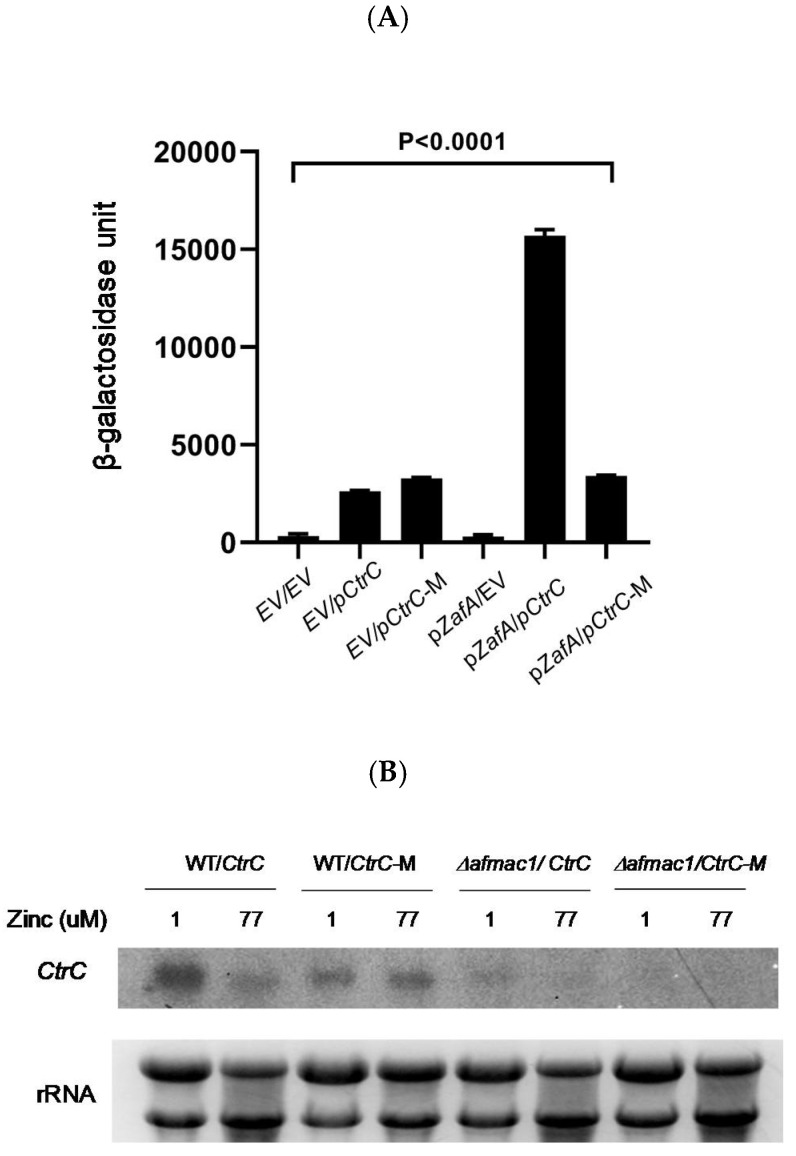
The ZafA binding motif has an important role in *CtrC* transcription induced by ZafA under zinc starvation conditions. To identify the role of the ZafA binding motif localized in the *CtrC* promoter region, β-galactosidase assays and Northern blotting were performed with a mutant ZafA binding motif. (**A**) The β-galactosidase assay was performed with a strain coexpressing *ZafA* and *CtrC*. *CtrC* and *ZafA* genes of *A. fumigatus* were subcloned into a yeast plasmid that encodes the LacZ reporter gene, and the yeast BY4741 strain was cotransformed with both plasmids. EV indicates empty vector, and *CtrC*-M indicates the mutant ZafA binding motif of the *CtrC* promoter region (Figure S1). Then, β-galactosidase activity was measured as described in materials and methods (*p* < 0.0001). (**B**) The expression of *CtrC* when the ZafA binding motif was mutated was investigated. A mutant strain of the ZafA binding motif of *CtrC* was constructed from wild-type and *Δafmac1* strains (Figure S2), and the indicated strains were cultured in media with different zinc concentrations (1 and 77 µM zinc). Northern blotting was performed to investigate the expression of *CtrC*.

To investigate the ability of ZafA to directly bind to the ZafA binding motif in *Ctr* genes, we performed an EMSA experiment. ZafA protein was purified from *E. coli* using a His-tagged form of ZafA, and overexpressed recombinant ZafA was used in the EMSA experiment. As shown in Figure 3, recombinant ZafA protein was incubated with amplified 118, 114, and 128 bp fragments of the ^32^P radiolabeled ZafA binding motif of *CtrA1*, *CtrA2*, and *CtrC*, respectively, and PAGE analysis was performed. Interestingly, ZafA strongly bound to the promoter region of *CtrC*, and a mobility shift was found, but did not bind to the promoter region *CtrA1* or *CtrA2*. Addition of 100-foldunlabeled cold ZafA binding motif of *CtrC* completely inhibited the mobility shift, and the specific band was found in the same region as the radiolabeled probe. To confirm the role of the conserved ZafA binding motif 5′-CAGGGT3′-3′ in the expression of *CtrC* genes, the *LacZ* reporter gene was fused with the upstream region, which included the conserved ZafA binding motif or a mutated form of the binding motif of *CtrC*, and p*LacZ*/*CtrC* and p*LacZ*/*CtrC*-M were constructed, respectively, as shown in Supplementary Figure S1. β-Galactosidase activity was then measured, as shown in Figure 4A. Yeast cells were cotransformed with pZafA and p*LacZ*/*CtrC* or p*LacZ*/*CtrC*-M, and β-galactosidase activity was measured. The p*LacZ*/*CtrC* and pZafA transformants showed higher β-galactosidase activity than those transformed with empty vector. However, the pZafA and p*LacZ*/*CtrC*-M transformants showed one-fifth the β-galactosidase activity of wild-type *CtrC*. This result indicates that the ZafA binding motif of *CtrC* has an important role in regulation of *CtrC* expression by ZafA under zinc starvation conditions, as shown in Figure 2.

### 2.4. ZafA Binds to the ZafA Binding Motif of the CtrC Promoter Region, and the Binding Affinity Is Inversely Related to Zinc and Copper Concentration

Furthermore, we performed Northern blot analysis with the mutant ZafA binding motif to investigate *CtrC* gene expression. The ZafA binding motif of *CtrC* was mutated from 5′-CAGGGT-3′ to 5′-GTCCCA-3′ at the chromosome level, as shown in Supplementary Figure S2, and the expression of CtrC was investigated. As shown in Figure 4B, *CtrC* gene expression was decreased by the increasing zinc concentration, and higher CtrC gene expression was observed at a low zinc concentration (1 µM) than at a high zinc concentration (77 µM). However, *CtrC* gene expression was not changed in the cells cultured in different zinc concentrations when the ZafA binding motif was mutated. Interestingly, the same result was found with the *Afmac1* deletion mutant. These results indicate that CtrC is regulated by zinc and ZafA in a manner independent of AfMac1. 

**Figure 5:**
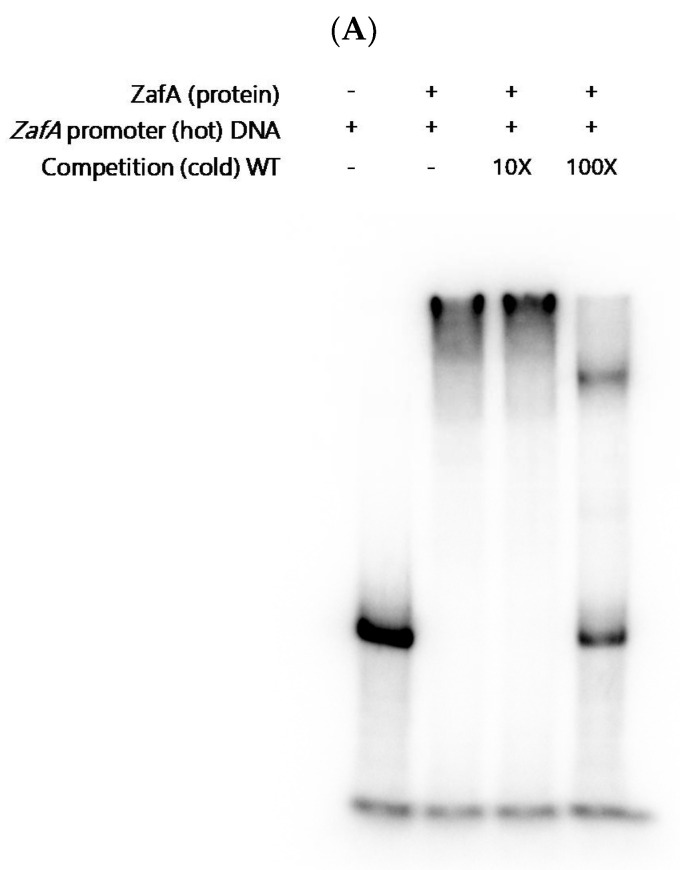

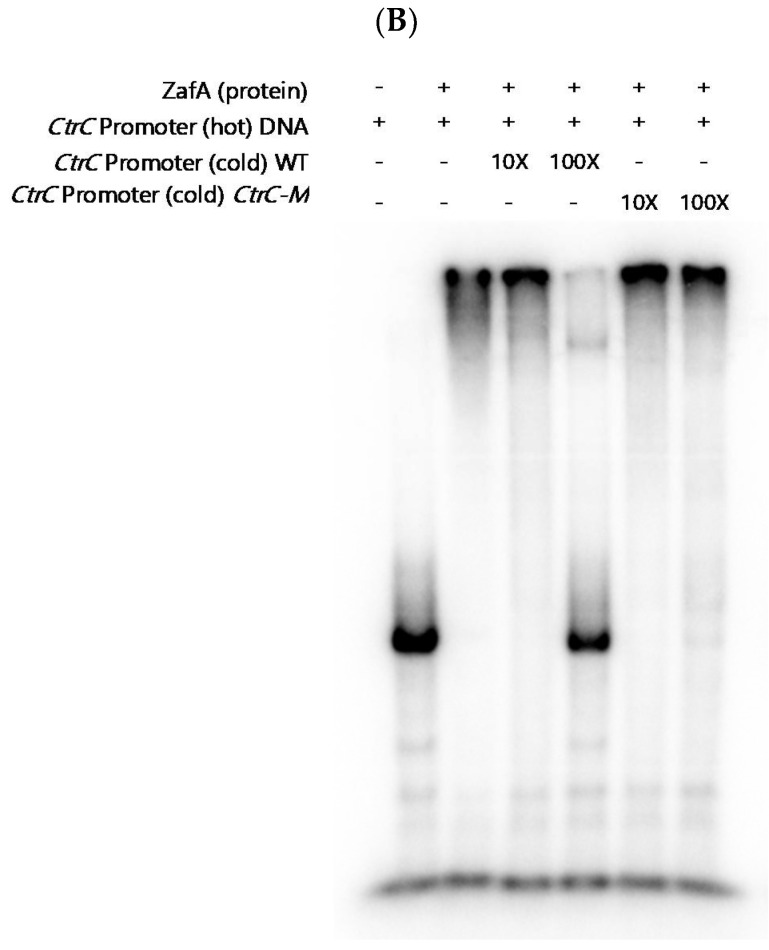
Mutation of the ZafA binding motif of *CtrC* led to failure of ZafA binding. To identify the binding affinity of ZafA to the ZafA binding motif of *CtrC*, an EMSA experiment was performed with a mutant ZafA binding motif of *CtrC*. (**A**) The recombinant ZafA strongly bound to the 95 bp length of the *ZafA* promoter region, and a cold probe acted as a competitor. (**B**) The binding affinity of ZafA to the mutant ZafA binding motif of *CtrC* was investigated. The cold wild-type ZafA binding motif and mutant form of the ZafA binding motif were used as competitors in the EMSA experiment.

To investigate the specificity of ZafA binding to the ZafA binding motif of *CtrC*, a mutated fragment of the ZafA binding motif of *CtrC* was used as a competitor. As shown in Figure 5B, the mutated ZafA binding motif of *CtrC* 5′-GTCCCA-3′ was used as a competitor, and an EMSA experiment was performed. The unlabeled, cold, wild-type ZafA binding motif of *CtrC* completely inhibited binding of ZafA to the ZafA binding motif of *CtrC*. However, the unlabeled mutated ZafA binding motif of *CtrC* failed to inhibit binding of ZafA to the ZafA binding motif of *CtrC*. This result indicates that ZafA binds to the ZafA binding motif of *CtrC* and regulates *CtrC* gene expression by specifically recognizing its conserved binding motif. 

**Figure 6:**
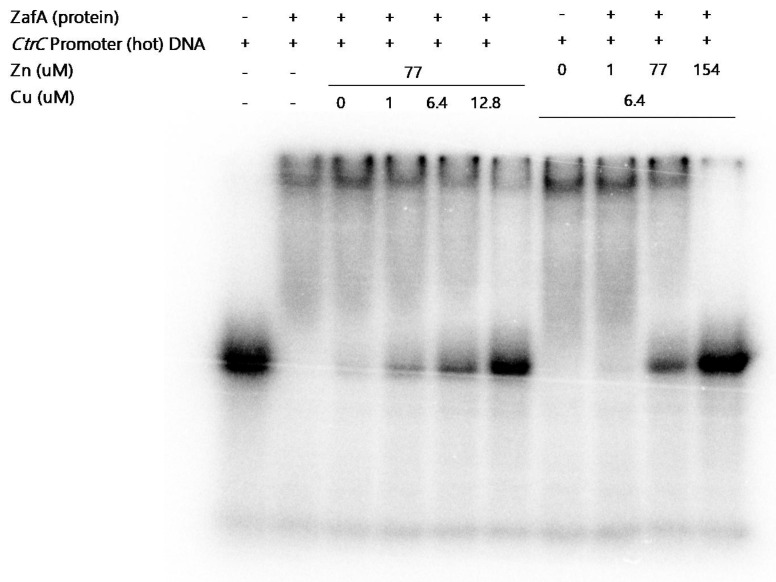
The binding affinity of ZafA to the ZafA binding motif of *CtrC* was inversely proportional to the copper or zinc concentration. To investigate the effect of zinc and copper on the binding affinity of ZafA to the ZafA binding motif of *CtrC*, the indicated zinc or copper concentration was used in the reaction mixture, and an EMSA experiment was performed.

Next, we examined the effect of zinc and copper on ZafA binding to the ZafA binding motif of *CtrC*. As shown in Figure 5A, we performed an EMSA experiment with the ZafA promoter region as a positive control and confirmed that recombinant ZafA works normally. We then performed the experiment to investigate the effect of zinc and copper on ZafA binding affinity to the *CtrC* promoter. As shown in Figure 6, ZafA bound to the ZafA binding motif of *CtrC,* and a mobility shift was observed. However, the mobility shift decreased with the zinc concentration, and 154 µM zinc inhibited the binding of ZafA to the ZafA binding motif of *CtrC*. Furthermore, we found that copper also partially inhibited binding of ZafA to the ZafA binding motif of *CtrC,* as shown in Figure 6. To further confirm the effect of zinc and copper on ZafA binding to the ZafA binding motif of *CtrC*, we performed an EMSA experiment under the standard zinc or copper concentration of AMM. The zinc concentration was fixed at 77 µM (the concentration in AMM), and the effect of copper was investigated. As shown in Figure 6, 12.8 µM copper completely inhibited ZafA binding to the ZafA binding motif of *CtrC* at 77 µM zinc, and a low concentration of copper activated ZafA binding to the ZafA binding motif. In addition, 154 µM zinc completely inhibited ZafA binding to the ZafA binding motif of *CtrC* at a copper concentration of 6.4 µM, which is the standard AMM concentration, and binding affinity was increased under a low zinc concentration. These results indicate that both zinc and copper regulate ZafA binding to the ZafA binding motif, especially at low concentrations, and suggest a physical interaction between zinc or copper and the ZafA protein to regulate ZafA activity.

## 3. Discussion

Acquisition of trace elements and their homeostasis in living cells are important for growth of microbial pathogens, and failure of metal homeostasis regulation results in loss of pathogenicity. Metals have diverse functions and work as a cofactors in physiological pathways, and iron, copper, and zinc perform important functions in living organisms. Recently, it has been reported that metals do not exert their function alone but interact with other metals and regulate homeostasis of other metals [20,41,42,43]. For example, our group previously reported that copper regulates iron metabolism and regulates gene expression involved in iron uptake in *A. fumigatus* [41]. There is a close interaction between copper and iron metabolism. 

Here, we have two questions, and the first is as to how zinc and copper interact with each other. Thus far, the functional interaction between copper and zinc metabolism has been studied in many groups, and it has been reported that copper and zinc compete for binding sites in target proteins and that zinc can induce copper deficiency and vice versa [44,45,46]. Thus, the copper and zinc concentration ratio is maintained in living organisms, and if the balance between copper and zinc is disrupted, it may cause health problems. Research groups have reported that the copper-to-zinc ratio is almost 2.0, and when the ratio is above 2.0, inflammation and oxidative stress are increased [47]. These reports indicate that competition between copper and zinc occurs in living organisms. Additionally, it has been reported that Wilson’s disease, which is a hereditary disorder caused by copper accumulation, can be alleviated by zinc therapy. That is, a high dose of zinc upregulates metallothionein, which binds copper, and copper detoxification occurs through excretion of copper via the bowels [48]. Additionally, a high zinc level restrains the copper level and disrupts the zinc/copper ratio in the body [47]. On the other hand, low dietary zinc changes copper functions in human beings [49]. These reports indicate that zinc and copper compete with each other, but the detailed working mechanism at the molecular level has not yet been reported. In this report, we investigated how zinc and copper interact at the molecular level using *A. fumigatus* as a model and identified the detailed mechanism by which zinc regulates copper metabolism. We found that zinc regulates copper uptake by regulating gene expression of copper transporters, especially *CtrC* of *A. fumigatus*. As shown in Figure 1, zinc starvation suppressed the *Δafmac1* phenotype, which grew slowly on AMM, and addition of zinc to the level present in AMM failed to suppress the *Δafmac1* phenotype. This result explained why zinc starvation suppressed the *Δafmac1* phenotype, and we investigated the detailed working mechanism. Interestingly, we found that expression of the high-affinity copper transporter *CtrC* is regulated by ZafA, which is a transcriptional activator of the genes involved in zinc metabolism. The conserved ZafA binding motif was found in the upstream region of *CtrC,* and mutagenesis of the ZafA binding motif failed to regulate *CtrC* expression induced by ZafA under a low zinc concentration. Furthermore, the regulation of *CtrC* gene expression by ZafA depends on copper utilization, as shown in Figure 4 and Figure 5. These results indicate that regulation of copper uptake by zinc is affected by copper concentration and support the interaction between copper and zinc. Interestingly, the results revealed that zinc regulates copper homeostasis but copper does not regulate zinc metabolism in *A. fumigatus*. 

The other question we wanted to answer is as to why the interaction between zinc and copper is necessary. We investigated the reason from the point of view of nutritional immunity in the fungal pathogen model system. Nutritional immunity is a host protection mechanism that inhibits the growth of pathogens and is carried out by limiting crucial nutrients of pathogens [34,35,50,51]. Nutritional immunity is observed in vertebrates and even plants [52,53]. Trace metals are the nutrients involved in nutritional immunity, and a host inhibits the growth of pathogens by limiting uptake of specific metal ions. Iron, copper, and zinc perform many physiological functions in virtually all living organisms and are necessary for growth. *A. fumigatus* is a representative fungal pathogen that infects immunocompromised human patients and has fatal effects. When *A. fumigatus* infects a host, neutrophils first attack the pathogen, and then zinc inside the neutrophils is eliminated by various metal transport systems to suppress fungal growth after ingestion of the pathogen [54]. By reducing the free zinc ion concentration inside neutrophils, a host can inhibit the growth of pathogens, and pathogens must look for other ways to survive. Our previous report showed that upregulation of gliotoxin production by zinc deprivation occurs in neutrophils [55]. In addition, upregulation of the metabolism of other metals is induced by zinc deprivation. In this report, we found that zinc starvation upregulates the expression of CtrC, which is a high-affinity copper transporter in *A. fumigatus.* Copper is also known as a virulence factor of *A. fumigatus,* and deletion of *AfMac1,* which is a transcription activator of the copper regulon, results in loss of virulence [11,14]. During infection, pathogens find a way to overcome host protection systems, and zinc starvation is a host protection system [9,56,57]. Upregulation of copper metabolism is a way to overcome zinc starvation caused by the host and may prolong the survival of pathogens. 

In this report, we explain the meaning of the interaction between zinc and copper in metal homeostasis, which will be helpful in understanding diseases caused by defective metal homeostasis in mammals. However, many questions still need to be answered to explain the relationship between different metals.

## 4. Materials and Methods

### 4.1. Strains, Media, and Plasmids

The fungal strain used in this study was *A. fumigatus* FGSC A1163 (*akuB* (KU80)-Δ*pyrG1*). The cells were grown in AMM (1% glucose, 70 mM sodium nitrate, 7 mM potassium chloride, 6 mM potassium phosphate, 5 mM MgSO_4_, and Hunter’s trace elements) or PD medium (7% infusion from potatoes and 2% dextrose). Medium was supplemented with 100 µM BCS (bathocuproinedisulfonic acid disodium salt, Sigma-Aldrich, St. Louis, MO, USA, 146625) for copper depletion. For generation of the *ΔzafA, Δafmac1*, and *ΔhapX* mutant strains, 5 flanking regions and 3 flanking regions of the respective gene were inserted into a *PyrG* blast cassette [11]. Each flanking region was amplified by PCR with a forward and reverse primer set: af.zafA 5flk F/R and 3flk F/R; af.mac1 5flk F/R and 3flk F/R; hapX 5flk F/R and hapX 3flk F/R. The amplified flanking regions were cloned into the *PyrG* fragment. To generate ZafA binding site mutants in the *CtrA1*, *CtrA2*, and *CtrC* promoter regions, mutation cassettes were constructed. The cassettes contained a mutated ZafA binding site in the promoter region. Each *Ctr* promoter region was amplified by PCR with a primer set: af.ctrC upst 1.6 kb F/700 bp R and af.ctrC upper F/5flk R; af.ctrA1 5flk F/R and af.ctrA1 3flk F/R; af.ctrA2 5flk F/R and af.ctrA2 3flk F/R. The wild-type genome was substituted with the mutated sequence in the chromosome. To express *CtrC* in the *Δafmac1* strain, a *CtrC* expression vector was constructed. *CtrC* was amplified by PCR with the primer set af.ctrC F/R, and then PCR-amplified *CtrC* was cloned into pPTRII-*PyrG* (this study). The plasmid was used to transform the auxotrophic *Δafmac1* strain via PEG-mediated protoplast transformation.

### 4.2. Site-Directed Mutagenesis

To generate the ZafA binding motif mutant in the *CtrA1*, *CtrA2*, and *CtrC* promoter region, 750 bp of the *CtrC* promoter region, 947 bp of the *CtrA1* promoter region, and 801 bp of the *CtrA2* promoter region were amplified by PCR with the following primer sets: af.ctrA1 5flk F/R, ctrA2 5flk F/R, and af.ctrC upper F/5flk R, respectively. Each promoter region was cloned into pGEMT-easy vector (Promega, Madison, WI, USA, A1360). The plasmid was used as the mutagenesis template. To mutate the ZafA binding motif from 5′-CAGGGT-3′ to 5′-GTCCCA-3′ or 5′-CAGGGTCAGGGT-3′ to 5′-GTTCCAGTTCCA-3′ in *CtrC, CtrA1*, and *CtrA2*, the following primer sets were used: af.ctrA1 ZafA BDM mutagenesis F/R, af.ctrA2 ZafA BDM mutagenesis F/R, and af.ctrC ZafA BDM mutagenesis F/R, respectively. Mutagenesis was performed with an EZchange Site-directed Mutagenesis kit (Enzynomics, Daejeon, Korea, EZ004) following the manufacturer’s protocol. Mutated sequences were confirmed by DNA sequencing analysis (BIONIX, Seoul, Korea).

### 4.3. Plate Assay

For plate assays, each strain was grown on an AMM plate for 3 days, and then fresh conidia were harvested with 0.01% Tween 80 solution. The conidia were filtrated with Miracloth (Millipore, Burlington, MA, USA, 475855). The wild-type strain was used as a control. Five hundred conidia were grown on AMM, AMM with 1 µM ZnSO_4_, AMM with 50 µM CuSO_4_, or AMM with 100 µM BSA and 1 µM ZnSO_4_ for 3 days at 37 °C.

### 4.4. Northern Blot Assay

For Northern blot assays, total RNA was isolated using RNAiso Plus (Takara, Shiga, Japan, 9108) according to the manufacturer’s protocol. The fungal cells were cultured in AMM or PD medium at 37 °C. Total RNA (10 µg) was separated on 1% formaldehyde–agarose gels via electrophoresis. The RNA was blotted onto Hybond-N membranes (GVS Life Sciences, Bologna, Italy, 1226556). P_32_ radioisotope labeling was performed with a DNA Labeling Kit (Takara, Shiga, Japan, 6045). DNA probes were amplified from *A. fumigatus* genomic DNA via PCR with a primer set: af.ctrA1 NP F/R, af.ctrA2 NP F/R, af.ctrB NP F/R, or af.ctrC NP F/R. The membrane was incubated in hybridization buffer (7% SDS, 1% BSA and 1 mM EDTA) with radioisotope-labeled DNA probes at 65 °C in a hybridization oven. The image was visualized on an imaging plate (Fujifilm, Tokyo, Japan, FLA-7000).

### 4.5. β-Galactosidase Assay

For β-galactosidase assays, 750 bp of the *CtrC* promoter region were amplified by PCR with the primer set af.ctrC 5flk F/R. The amplified promoter region was cloned into pGEMT-easy vector. Then, the *CtrC* promoter region was digested with *Spe*I and *BamH*I, and the digested DNA fragment was ligated with *LacZ* fragments. The *LacZ*-fused CtrC promoter was cloned into the yeast vector pRS425. *ZafA* was amplified by PCR with the primer set af.zafA XhoI F/BamHI R from fungal cDNA. The *ZafA* cDNA was digested with XhoI and BamHI and subcloned into the yeast vector pYPEG15. Wild-type BY4741 yeast cells were cotransformed with both plasmids. β-Galactosidase assays were performed according to the following method: The transformants were grown in SD-LU broth until reaching an optical density of approximately 0.6–0.8. The yeast was harvested and resuspended in Z-buffer (16.1 g/L Na_2_HPO_4_∙7H_2_O, 5.5 g/L NaH_2_PO_4_∙H_2_O, 0.75 g/L KCl, 0.246 g/L MgSO_4_∙7H_2_O, pH 7.2). The cells were frozen in liquid nitrogen and thawed at 37 °C in a water bath 5 times. ONPG/Z-buffer (4 mg/mL of ONPG in Z-buffer) was added and incubated with the cells at 30 °C until the color turned yellow. The OD was measured at 420 and 600 nm. β-Galactosidase units were calculated using the formula described by Miller (1972, Yeast Protocol Handbook, Takara, Shiga, Japan, PT3024-1, 27-28).

### 4.6. ZafA Protein Purification

For ZafA purification, *ZafA* cDNA in pGEMT-easy vector was digested with *BamH*I and *Xho*I and then cloned into the *E. coli* vector pET-21(+). His-tagged ZafA was generated in *E. coli* BL21. ZafA expression was induced by 0.1 M IPTG at 4 °C overnight. The cells were lysed in lysozyme lysis buffer (50 mM NaH_2_PO_4_, 50 mM NaCl, 1 mM EDTA, 1 mM PMSF, and 2 µg/mL lysozyme), and the His-tagged ZafA was purified using Ni-NTA agarose resin (Qiagen, North Rhine-Westphalia, Germany, 30210). The ZafA concentration was measured with a Bradford assay (Biorad, Hercules, CA, USA, 5000002).

### 4.7. Electrophoretic Mobility Shift Assay (EMSA)

For EMSA assays, DNA probes that contained the ZafA binding motif upstream of the *ZafA* or *Ctr* genes were designed. Each DNA probe was amplified by PCR with a specific primer set: af.zafA 5flk EMSA F/R, af.ctrA1 EMSA F/R, af.ctrA2 EMSA F/R, or af.ctrC EMSA F/R. The amplified probes were labeled with P_32_ radioactive isotope using *Bst* DNA polymerase (Enzynomics, Daejeon, Korea, DP004). Next, 500 ng of ZafA protein and 5 ng of P_32_-labeled probes were incubated in binding buffer (4% glycerol, 1 mM MgCl_2_, 500 µM EDTA, 500 µM DTT 50 mM NaCl, 10 mM Tris HCl pH 8.0) with or without unlabeled competitors (cold DNA) for 30 min at 25 °C. To investigate the effect of zinc or copper on ZafA binding to *Ctr* genes, zinc and copper were added 10 min after ZafA and DNA binding was started. The protein–DNA complex was resolved in a Tris–boric acid–EDTA (TBE)-based 8% native acrylamide gel via electrophoresis. The gel was dried on 3 M paper, and the shifted image was visualized on an imaging plate (Fujifilm, Tokyo, Japan, FLA-7000).

## Supplementary Materials

Supplementary materials can be found at https://www.mdpi.com/1422-0067/21/20/7665/s1.
