# Supplementary material for: The Role of Zinc in Copper Homeostasis of Aspergillus fumigatus
Source: Int J Mol Sci. 2020 Oct 16;21(20):7665. doi: 10.3390/ijms21207665 (PMC7593903; doi:10.3390/ijms21207665)
Supplement: Supplementary file 1 [file ijms-21-07665-s001.zip › ijms-949923-supplementary.docx]

**Supplementary data**

**Figure S1.** To construct the LacZ reporter gene, 750 bp of the *CtrC* promoter region containing the wild-type ZafA binding motif (5’-CAGGGT-3’) or mutated sequence (5’-GTCCCA-3’) were amplified by PCR with the primer pair af.ctrC 5flk F and af.ctrC 3flk R and then digested with *Spe*I and *BamH*I. Digested DNA fragments were ligated with *LacZ* fragments. Each *LacZ*-conjugated *CtrC* promoter region was digested with *Spe*I and *Hind*III and introduced into the yeast vector pRS425. Red indicates the ZafA binding motif.

**Figure S2.** To construct the mutant ZafA binding motif in the *CtrC* promoter region on the chromosome, 750 bp of the *CtrC* promoter region containing the wild-type ZafA binding motif (5’-CAGGGT-3’) or mutated sequence (5’-GTCCCA-3’) were amplified by PCR, and then, the mutant strain was constructed as described in materials and methods. Red indicates the ZafA binding motif.

**Table S1.** The primers used in this study.

| **Primer name** | **Sequence 5' to 3'** |
| --- | --- |
| af.ZafA 5UTR F | GGTAAGCGGATCGGAGGATA |
| af.ZafA 5UTR R | GAAGATCCGCTAGCAATGGG |
| af.ZafA 3UTR F | CATTGGAGTTGGACAAGGCG |
| af.ZafA 3UTR R | CAAACCTCTACCCTCCCGTA |
| af.mac1 5flk F | TTCCTGAAGCTAGTCGCAGC |
| af.mac1 5flk R | TGGGAATCTCAACTCAACCTAGA |
| af.mac1 3flk F | TGCCTTTTACTGCACATCGC |
| af.mac1 3flk R | GGCTGACACGGTTCTAGCTT |
| af.HapX 5UTR F | TTCGAGGTAAGGAAGCCTGTC |
| af.HapX 5UTR R | ATCTGGATGGACATTAGTGGGG |
| af.HapX 3UTR F | ATACCCAAACGGACTCGTGA |
| af.HapX 3UTR R | AGTCATTCTGCGTTCGGAGC |
| af.ZafA 1168 F | AACCATATGGGGACTGTGGA |
| af.ZafA StopX Xho1 R | CTCGAGAACAGTGCCAAGGG |
| ZafA NP F | AAGATGATTTCTGCCTCGAA |
| ZafA NP R | CAGCATTGAGTCTAGATTGT |
| af.ctrA1 NP F | CGTGATGACGTTCAATGTG |
| af.ctrA1 NP R | CATCCTGCCATCCTAATGAA |
| af.ctrA2 NP F | GAATCAGTAAAGGTTGTGCG |
| af.ctrA2 NP R | CGCCTGTAATACACAGGAAT |
| af.ctrB NP F | ATCATCCTTGCAGCTTTGTA |
| af.ctrB NP R | AAGCATGACGAACCCATTAT |
| af.ctrC NP F | TCCATGACTTCAACCATGAC |
| af.ctrC NP R | TATCCATGCCACCCATACTA |
| af.zafA 5flk EMSA F | TACCAGGGCCCCTTTCCCTCAA |
| af.zafA 5flk EMSA R | ATCCAAGAGGACCTTGGGAGCTT |
| af.ctrA1 EMSA F | TGCCGCCATTGTTCAGCAGTGC |
| af.ctrA1 EMSA R | TCGGATCGTTGCCGAGGAAATGAT |
| af.ctrA2 EMSA F | CTCAAAGCTCGCTCATCGGA |
| af.ctrA2 EMSA R | AGTAAGAATGCTACGCCGCC |
| af.ctrC EMSA F | AAGGGCCCAGGGCCTGTCTTG |
| af.ctrC EMSA R | AGCAGACCAGAGCAATAGAAGAAAGGGT |
| af.ctrC 5flk F | ATGTTAATGTTCTGGGGGCCAA |
| af.ctrC 5flk R | TTCTGGTTGGATAGACGATTGTA |
| af.ctrC 5flk upper F | TATCTACTTGACATCATGTGA |
| af.ctrC upst 1.6 kb F | AGCGCAGATTTCTACCAACCC |
| af.ctrC upst 700 bp R | TCATACTCCTCGGTTTGTCCCCT |
| af.ctrA2 5flk F | ATGTCTGCGGTGTCGAAGAG |
| af.ctrA2 5flk R | GTCTGTTGTCTATGTATATTGTCCTGA |
| af.ctrA1 5flk mutagenesis F | CCATTGTTCAGCAGTGCGGTGCTGTCAAGGACGGCCTTCT |
| af.ctrA1 5flk mutagenesis R | AGAAGGCCGTCCTTGACAGCACCGCACTGCTGAACAATGG |
| af.ctrA2 5flk mutagenesis F | GTGACAGAGCATTGAGAATGTGAAGAAT |
| af.ctrA2 5flk mutagenesis R | ATTCTTCACATTCTCAATGCTCTGTCAC |
| af.ctrC 5flk mutagenesis F | ACTTCACTTTCTCCGTCCCAGCCACTGCTTGCTA |
| af.ctrC 5flk mutagenesis R | TAGCAAGCAGTGGCTGGGACGGAGAAAGTGAAGT |
